# Patient Acceptability of Symptom Screening and Patient Education Using a Chatbot for Autoimmune Inflammatory Diseases: Survey Study

**DOI:** 10.2196/49239

**Published:** 2023-12-28

**Authors:** Tze Chin Tan, Nur Emillia Binte Roslan, James Weiquan Li, Xinying Zou, Xiangmei Chen, Ratnasari, Anindita Santosa

**Affiliations:** 1 Department of Rheumatology and Immunology Singapore General Hospital Singapore Singapore; 2 Medicine Academic Clinical Programme SingHealth-Duke-NUS Singapore Singapore; 3 Department of General Medicine Sengkang General Hospital Singapore Singapore; 4 Department of Gastroenterology and Hepatology Changi General Hospital Singapore Singapore; 5 Internal Medicine Clinic Changi General Hospital Singapore Singapore; 6 Division of Rheumatology and Immunology, Department of Medicine Changi General Hospital Singapore Singapore

**Keywords:** conversational agents, digital technology in medicine, rheumatology, early diagnosis, education, patient‒physician interactions, autoimmune rheumatic diseases, chatbot, implementation, patient survey, digital health intervention

## Abstract

**Background:**

Chatbots have the potential to enhance health care interaction, satisfaction, and service delivery. However, data regarding their acceptance across diverse patient populations are limited. In-depth studies on the reception of chatbots by patients with chronic autoimmune inflammatory diseases are lacking, although such studies are vital for facilitating the effective integration of chatbots in rheumatology care.

**Objective:**

We aim to assess patient perceptions and acceptance of a chatbot designed for autoimmune inflammatory rheumatic diseases (AIIRDs).

**Methods:**

We administered a comprehensive survey in an outpatient setting at a top-tier rheumatology referral center. The target cohort included patients who interacted with a chatbot explicitly tailored to facilitate diagnosis and obtain information on AIIRDs. Following the RE-AIM (Reach, Effectiveness, Adoption, Implementation and Maintenance) framework, the survey was designed to gauge the effectiveness, user acceptability, and implementation of the chatbot.

**Results:**

Between June and October 2022, we received survey responses from 200 patients, with an equal number of 100 initial consultations and 100 follow-up (FU) visits. The mean scores on a 5-point acceptability scale ranged from 4.01 (SD 0.63) to 4.41 (SD 0.54), indicating consistently high ratings across the different aspects of chatbot performance. Multivariate regression analysis indicated that having a FU visit was significantly associated with a greater willingness to reuse the chatbot for symptom determination (*P*=.01). Further, patients’ comfort with chatbot diagnosis increased significantly after meeting physicians (*P*<.001). We observed no significant differences in chatbot acceptance according to sex, education level, or diagnosis category.

**Conclusions:**

This study underscores that chatbots tailored to AIIRDs have a favorable reception. The inclination of FU patients to engage with the chatbot signifies the possible influence of past clinical encounters and physician affirmation on its use. Although further exploration is required to refine their integration, the prevalent positive perceptions suggest that chatbots have the potential to strengthen the bridge between patients and health care providers, thus enhancing the delivery of rheumatology care to various cohorts.

## Introduction

### Background

Digital health technologies, including chatbots and conversational artificial intelligence (AI) agents, have the potential to reshape health care delivery and patient outcomes [1,2]. Chatbots use natural language processing and AI to mimic human interactions and foster efficient communication between patients and health care providers. These digital tools can potentially transform medical care by aiding in patient screening, triage, and education, while simultaneously alleviating the strain on health care systems [3].

Although chatbots can potentially transform chronic disease management, evidence of their sustained adoption remains limited, particularly for autoimmune conditions [4,5]. Currently, most chatbot applications focus on oncology and mental health, with minimal customization for inflammatory rheumatic diseases [6,7]. Early diagnosis and treatment of inflammatory rheumatic diseases are critical for preventing irreversible joint damage, disability, and other complications [8]. Unfortunately, significant barriers, including rheumatologist shortages, high costs, and low public awareness, restrict optimal rheumatic care globally. Chatbots can address these challenges by facilitating patient education, self-triage, and access to rheumatology expertise. Despite the immense potential of chatbots to improve rheumatic disease management, there is minimal research on their real-world implementation and efficacy.

To address this gap, we evaluated patients’ perceptions and acceptance of a chatbot purpose-built for rheumatology. We aimed to provide insights into the successful adoption of chatbots in rheumatology care by assessing user attitudes, satisfaction, demographics, and the differences between the first and follow-up (FU) encounters. In the following section, we review the literature on conversational AI agents in health care, focusing on autoimmune inflammatory rheumatic diseases (AIIRDs). We then present the methodology and results of a survey evaluating patients’ acceptance of an AIIRD chatbot in a rheumatology clinic. Finally, we discuss the implications of our findings, limitations, and the future research needed to realize the potential benefits of thoughtfully designed chatbots for improving outcomes in autoimmune diseases.

### Literature Review

#### Conversational AI Agents in Health Care

Chatbots can facilitate early disease detection and streamline referral processes, ultimately contributing to better patient outcomes [9,10]. They show considerable promise in increasing efficiency, reducing costs, and promoting patient satisfaction in various health care settings [11]. Patients are likely to benefit from prompt and accurate information provided by chatbots, thereby enhancing their overall care experience [8,12]. Notable examples include OneRemission (Keenethics), which provides patients with cancer with detailed health insights; Youper (Youper Inc), a personalized conversational assistant dedicated to mental well-being support [13-15]; Babylon Health (eMed Healthcare UK), known for its symptom checking and virtual consultations; and Engati (Engati) and Inbenta (Inbenta) chatbots, which have the potential to manage a range of patient inquiries and provide essential medical information.

The usefulness of digital health care technology has been the subject of an expanding body of research, including how well it performs when used for patient screening and classification during the COVID-19 pandemic [16,17]. Existing evidence indicates that the adoption and acceptability of chatbots in health care are subject to a multitude of technological, socioeconomic, and linguistic barriers. Consequently, despite the considerable potential of chatbots, their acceptability in health care settings remains inconsistent across patient demographics [18-22]. It has been reported that subjective norms, health awareness, and perceived convenience affect attitudes toward medical chatbots [23], and chatbot designers should focus on a user-centered framework and legal implications to address patient concerns. Moreover, chatbots, similar to any digital technology, may pose data privacy concerns, particularly given the sensitive nature of health-related information, and the accuracy of chatbot responses governed by their underlying algorithms can vary [24,25]. Misinterpretation of user input or delivery of inaccurate health advice can lead to suboptimal patient care or health risks. Thus, while chatbots offer promising possibilities for enhancing health care delivery, they should be used judiciously and continually optimized to ensure safety, accuracy, and respect for user privacy.

#### Conversational AI Agents in AIIRDs

Preliminary evidence indicates that chatbots could play a substantial role in the screening and triaging of rheumatic diseases, emphasizing their potential contribution to this specialized area of medical practice. Unfortunately, few studies have reported results or patient perspectives on chatbots used in the context of chronic diseases [8,12]. To address the challenges in AIIRD identification and prioritization, health care systems must implement innovative strategies that improve screening and triage processes for patients with AIIRDs [26-28]. To this end, digital technologies such as telemedicine, electronic health records, and AI algorithms have been proposed to improve early diagnosis and streamline the referral process [29].

#### Implementing Conversational AI Agents

The ultimate efficacy of chatbot implementation depends on user acceptability and perceived value [9]. Implementation science is the study of methods and strategies to promote the systematic acceptance of research findings in routine clinical practice to improve the quality and effectiveness of health care. It is grounded in several theoretical frameworks, including the Consolidated Framework for Implementation Research and the RE-AIM (Reach, Effectiveness, Adoption, Implementation and Maintenance) framework [30,31]. These frameworks provide a comprehensive approach for studying the implementation of health care interventions and can help identify the factors that influence adoption and implementation.

## Methods

### Definitions

#### About AIIRD

AIIRDs include rheumatoid arthritis, psoriatic arthritis, ankylosing spondylitis, and systemic lupus erythematosus. They are characterized by aberrations in the immune system that lead to chronic inflammation in various parts of the body, particularly the joints.

#### Chatbot

A chatbot is software designed to interact with humans in their natural language. These interactions usually occur through messaging applications, websites, mobile apps, or over the phone. In health care, chatbots can provide valuable assistance by answering patient queries, helping with diagnoses, or providing medical information.

### RE-AIM Framework

The RE-AIM framework serves as a robust model for evaluating the impact of health interventions encompassing 5 pivotal dimensions: reach, effectiveness, adoption, implementation, and maintenance [32,33]. In our study, we used this framework to design a survey to meticulously assess the effectiveness, acceptability, and implementation processes of chatbots in health care settings. Specifically, the survey, detailed in Table 1, was constructed to scrutinize the various facets of participants’ interactions with the chatbot, including its effectiveness, user-friendliness, and potential applicability. The survey questions were structured to resonate with the dimensions of the RE-AIM framework, establishing a coherent link between the survey instrument and the framework, as elucidated below.

**Table 1:** Chatbot acceptance survey.

Number	Question	Response options
Q1	Was the chatbot able to answer your question satisfactorily?	1 strongly disagree to 5 strongly agree
Q2	Was the chatbot easy to navigate?	1 strongly disagree to 5 strongly agree
Q3	Was the information presented easy to understand?	1 strongly disagree to 5 strongly agree
Q4	I am comfortable with the use of chatbots to look up information before my consultation	1 strongly disagree to 5 strongly agree
Q5	I am comfortable with the use of chatbots to look up information after my consultation	1 strongly disagree to 5 strongly agree
Q6	I will use the chatbot again if it allows me to find the cause of my symptoms and how to manage them	1 strongly disagree to 5 strongly agree
Q7 BC^a^	I am comfortable with the idea of a chatbot making a diagnosis based on your symptoms (before consultation)	1 strongly disagree to 5 strongly agree
Q7 AC^b^	I am comfortable with the idea of a chatbot making a diagnosis based on your symptoms (after consultation)	1 strongly disagree to 5 strongly agree

^a^BC: before the consultation.

^b^AC: after the consultation.

### Effectiveness Dimension

Q1 evaluated the chatbot’s prowess in responding satisfactorily to user inquiries, thereby gauging its effectiveness in addressing diverse concerns. Q3 assessed the comprehensibility of the information rendered by the chatbot to ensure effective interpretation and application in health care decisions. Q5 probed participants’ comfort in using chatbots for information retrieval after consultation, shedding light on their supplementary informational value. The categorization of rheumatological diagnoses into 8 groups measured the chatbot’s impact on disease screening and triaging, aligned with the effectiveness dimension.

### Adoption Dimension

Q2 measured the ease of navigation within the chatbot, which is a critical factor that influences user satisfaction and chatbot adoption feasibility. Q6 gauged participants’ willingness to engage with the chatbot in the future, especially if it aids in symptom identification and offers effective management guidance.

### Reach Dimension

Q4 investigated participants’ comfort in using chatbots for pertinent information retrieval before the consultation, emphasizing chatbots’ potential in the preconsultation phase.

### Implementation Dimension

Q7, posed before the consultation (BC) and after the consultation (AC), explored participants’ comfort levels regarding chatbots making diagnoses based on their symptoms, unveiling any attitudinal shifts toward the chatbots’ diagnostic role after the consultation.

### Participants and Study Design

A pilot study was conducted from June to October 2022, with a predetermined sample size of 200 participants, which included 100 new cases and 100 FU cases. The sample size was determined based on previous studies [34,35]. Upon registration in the rheumatology outpatient clinic, a random sample of patients aged ≥17 years with a diagnosis of AIIRD or a referral for suspected AIIRD was selected. The participants were invited to participate in the survey, and verbal consent was obtained before administering the survey questions. Patients diagnosed by rheumatologists were classified into 3 groups: arthropathies, connective tissue diseases or vasculitides, and other diagnoses. Patient visits were classified into 2 types: first visit (FV), indicating the initial clinic visit, and FU visit, indicating return visits after the initial visit. The patient education level was classified as below university education, university degree, or higher.

### Chatbot: SingHealth RheumConnect

**Figure 1:**
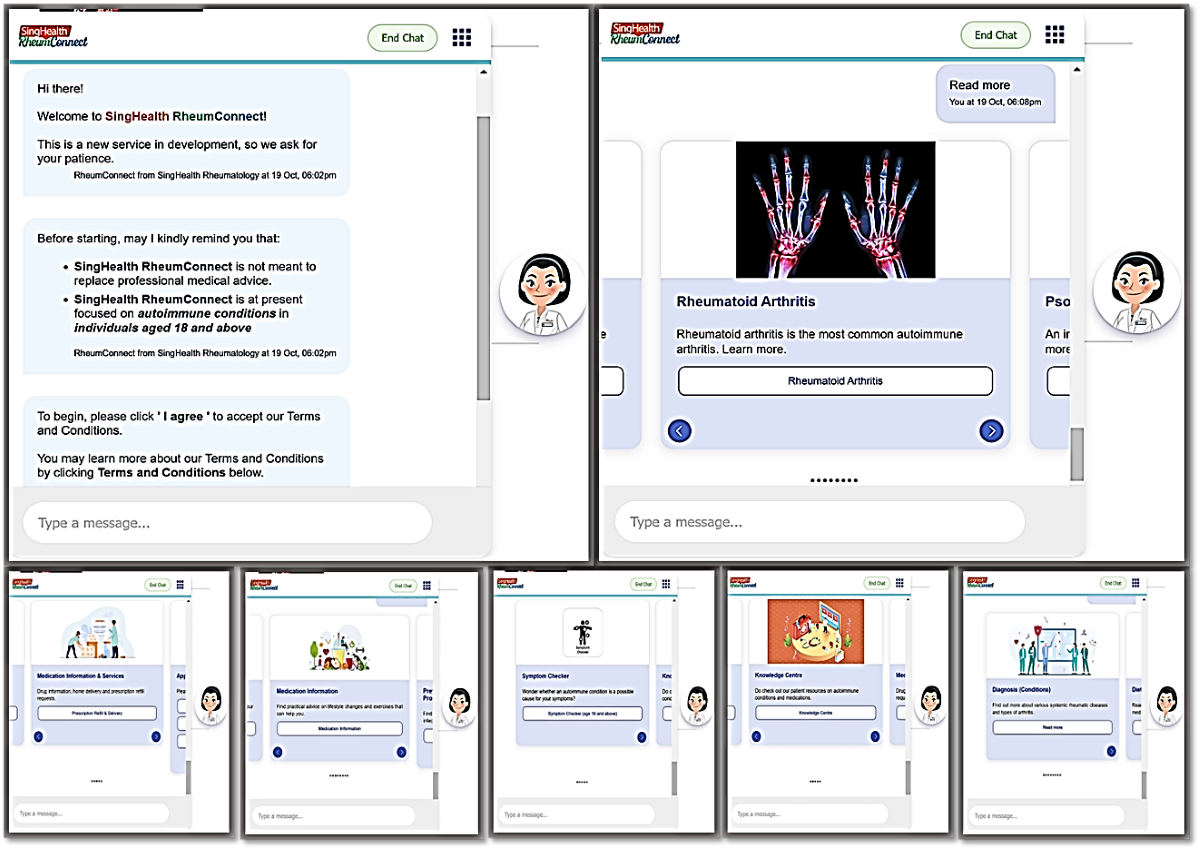
Representative screenshots of the chatbot interface.

SingHealth RheumConnect is a rule-based chatbot designed for internet-enabled devices. It was introduced in 2022 and serves as a valuable web-based resource for patients seeking treatment for rheumatology. The chatbot aims to alleviate resource and service deficiencies in rheumatology care by facilitating referrals and aiding patients in symptom management. Figure 1 and Figures S1-S7 in Multimedia Appendix 1 show the representative screenshots of the interface. SingHealth RheumConnect offers comprehensive information regarding various rheumatic diseases and arthritic conditions. The conditions covered were gout, systemic lupus erythematosus (lupus), myositis, osteoarthritis, psoriatic arthritis, rheumatoid arthritis, spondyloarthritis, systemic sclerosis, and vasculitides. This platform serves patients, caregivers, and the public. SingHealth RheumConnect also features an intuitive symptom checker to assess whether symptoms could indicate an autoimmune disease. It also connects users to essential services such as prescription renewals and appointment modifications. The chatbot is now being incorporated for normal use and is available 24/7 at no cost. Personal information is not collected during the advice-seeking interactions.

### Statistical Analysis

Statistical analyses were performed using Excel (Microsoft Excel 365, Microsoft Corporation) and SPSS software (version 28.0, IBM SPSS Statistics for Windows). Independent sample 2-tailed *t* tests or 1-way ANOVA were used to compare continuous variables, such as age and questionnaire scores. Chi-square tests were used to compare categorical variables such as sex, education, and diagnosis categories between the groups. A paired *t* test was conducted to compare the attitudes of the participants regarding the acceptability of chatbot diagnoses before and after clinical consultation. Specifically, this analysis compared the mean scores on the questionnaire items Q7 BC and Q7 AC. Q7 asked about comfort with the idea of a chatbot making a diagnosis based on the reported symptoms.

Linear regression analysis was performed to assess the factors associated with the Q6 score, which showed a significant difference between the FV and FU visit. Q6 asked about the intent to use the chatbot again if it helped determine the causes of the symptoms. The predictors entered into the regression model included sex (male or female), age (continuous), type of visit (FV vs FU), education (below university vs university degree or above), and diagnostic category (arthropathies vs connective tissue diseases or vasculitides vs other diagnoses). Q6 score was treated as a continuous dependent variable. Univariate linear regression was performed for the first time to examine the unadjusted associations between each predictor and Q6 score. Variables with a *P* value <.25 were selected for the final multivariate model entry. Multivariate linear regression using the enter method was conducted to evaluate the independent predictors of the Q6 score after adjusting for other variables in the model. Beta coefficients with 95% CIs were estimated. The variance inflation factor was examined to assess multicollinearity between predictors. Statistical significance was set at *P*<.05.

### Ethical Considerations

No written consent or ethical approval was obtained because the collected information did not include any personal data, thus not allowing retrospective identification of the survey participants.

## Results

This study included 200 participants with the same number of FV and FU cases (Table 2). Female participants predominated the sample (n=121, 60.5%), and most of the participants (n=120, 60%) were university educated. A comparison between the FV and FU groups revealed notable differences in the patient characteristics. The FV group had a lower mean age (42.52, SD 13.80 y) than the FU group (54.35, SD 23.04 y), resulting in an overall mean age of 48.44 (SD 19.85) years for the entire sample (*P*<.001). However, the FV and FU groups did not show significant differences in the education level or diagnostic category (both *P*>.05).

**Figure 2:**
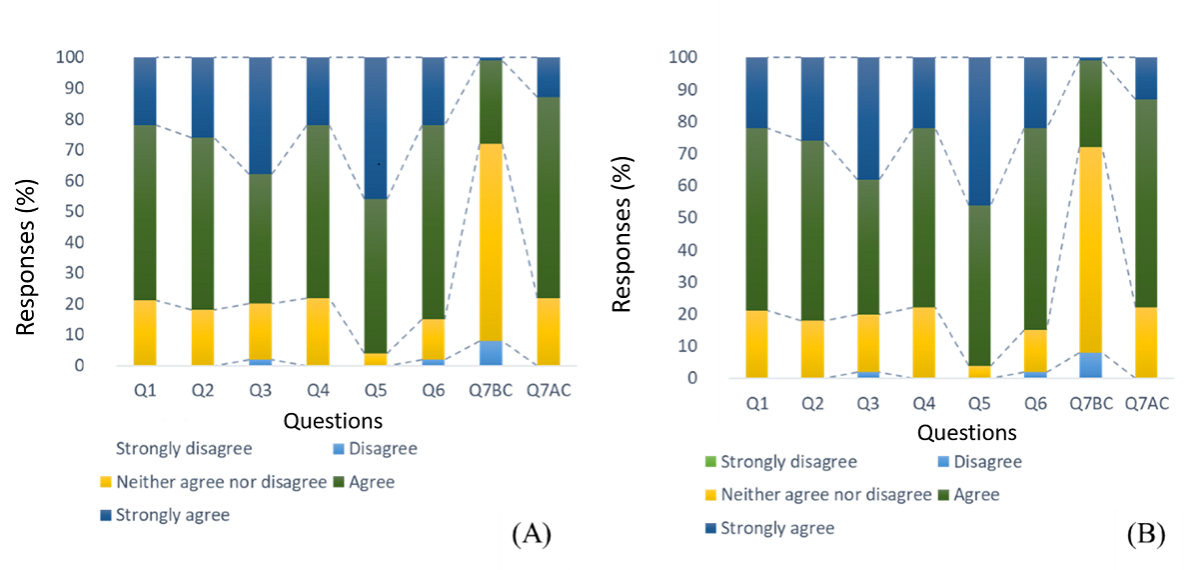
Patient responses to the SingHealth RheumConnect chatbot interaction: (A) FV and (B) FU visits. The x-axis illustrates the range of questions, while the y-axis indicates the percentage of responses across different rating scales. The evaluation of chatbot experience was derived from the questions listed in Table 1 and discussed in the Methods section. Most FV and FU patients agreed that the chatbot provided satisfactory responses, displayed user-friendliness, and conveyed information in a comprehensible manner. AC: after the consultation; BC: before the consultation; FU: follow-up; FV: first visit; Q1-Q7: questionnaire items that assess patient attitudes.

**Figure 3:**
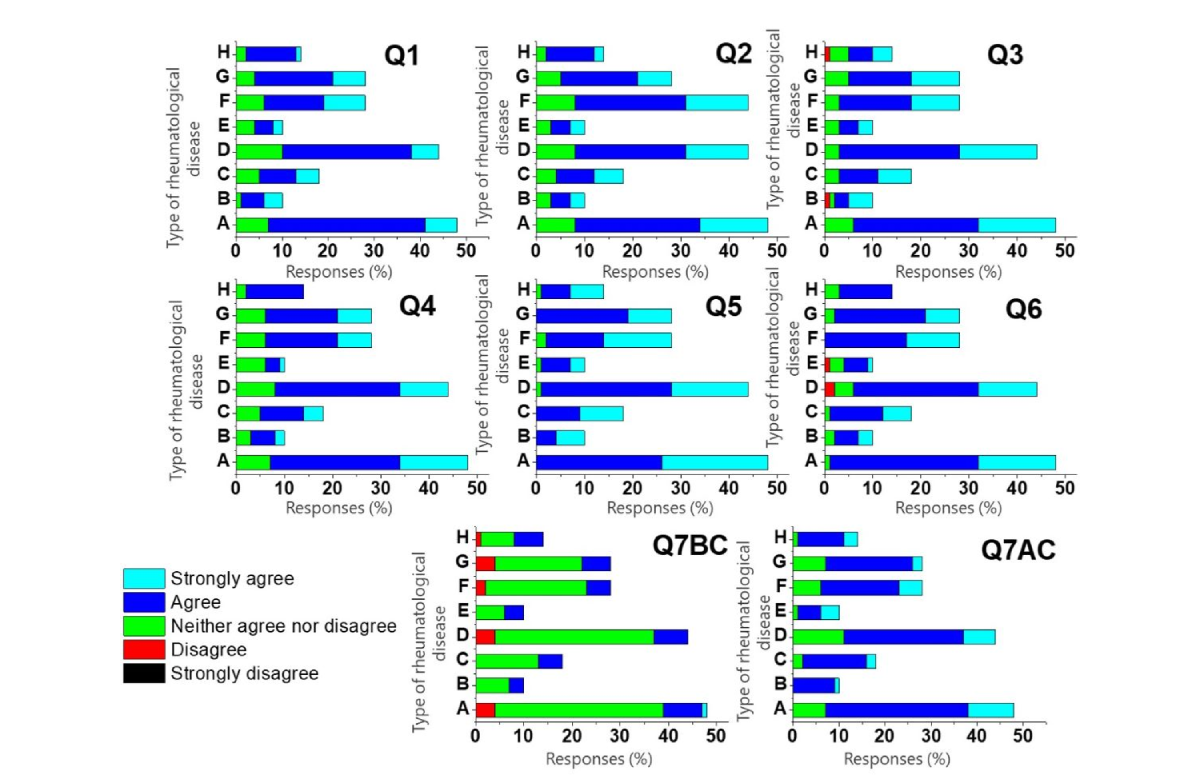
The outcome of the SingHealth RheumConnect chatbot acceptability survey for patients seeking information on different diseases, including (A) crystal arthropathy (n=48), (B) osteoarthritis (n=10), (C) psoriatic arthritis (n=18), (D) rheumatoid arthritis (n=44), (E) spondyloarthropathies (n=10), (F) SLE (n=28), (G) other CTD and vasculitides (including overlap syndromes; n=28), and (H) others (n=14). The y-axis represents the type of rheumatological disease, while the x-axis indicates the percentage of responses across different rating scales for chatbot acceptability. AC: after the consultation; BC: before the consultation; CTD: connective tissue disease; Q1-Q7: questionnaire items that assess patient attitudes; SLE: systemic lupus erythematosus.

Figure 2 shows that no participant selected the “strongly disagree” or “disagree” option. In the FV group, out of 100 participants, 21 (21%) remained neutral, with a response of “neither agree nor disagree.” Similar sentiments were found among 18 (18%) out of 100 participants in the FU group. Interestingly, only 2 (2%) out of 100 participants from the FV group expressed concerns about the clarity of the information in Q3, whereas the FU group had no such reservations. Figure 3 further illustrates the widespread acceptance of chatbots across the various diagnostic categories.

When evaluating the efficacy of the chatbot in addressing user queries (Q1), both the FV and FU groups showed a consistent mean score of 4.01 (SD 0.63), as shown in Table 2. The navigability of the chatbot (Q2) also received positive feedback, with an average score of 4.09 (SD 0.69) in both groups. The participants were confident in the clarity of the chatbot information, as indicated by Q3, which showed mean scores of 4.16 (SD 0.79) and 4.23 (SD 0.62) for the FV and FU groups, respectively, culminating in an overall mean of 4.20 (SD 0.71).

The comfort of using the chatbot before and after the consultations, as inferred from Q4 and Q5, exhibited consistent means across both groups. Specifically, for the FV and FU groups, Q4 recorded means of 4.00 (SD 0.67) and 4.02 (SD 0.67), respectively, and Q5 achieved means of 4.42 (SD 0.57) and 4.39 (SD 0.51), respectively, reflecting a high degree of comfort among participants. However, responses to Q6, which delved into the chatbot’s ability to discern symptom causes, showed a more pronounced mean score among FU participants at 4.29 (SD 0.57) than the FV participants who scored it at 4.05 (SD 0.66). The overall mean score for this question was 4.17 (SD 0.63).

The participants’ responses indicated different feelings about the chatbot making a diagnosis before and after consultation with the physician. Specifically, when asked if they would be comfortable with the chatbot making a diagnosis before meeting the physician (Q7 BC), participants were neutral, with mean scores of 3.21 (SD 0.59; FV group) and 3.10 (SD 0.48; FU group) on a 5-point scale. However, participants reported feeling significantly more comfortable having the chatbot make a diagnosis after meeting with the physician (Q7 AC; *P*=.04), with higher mean scores of 4.08 (SD 0.58; FV group) and 3.91 (SD 0.59; FU group). A paired *t* test confirmed that participants were significantly more at ease with the chatbot making a diagnosis before the consultation than after the consultation (*P*<.001).

The responses to the questionnaire did not differ significantly by sex, education level, or diagnosis category, as shown in Tables S1-S3 in Multimedia Appendix 1. However, as shown in Table 2, a noticeable difference was observed in the responses to questionnaire item Q6 between the FV and FU groups (*P*=.006). Multivariate linear regression was used to identify the factors that influenced this difference. The variables, resulting regression coefficients, and 95% CI are presented in Tables 3 and 4. Sex did not correlate significantly with responses to Q6; male participants had a coefficient of 0.066 (95% CI –0.115 to 0.247; *P*=.47) compared to female participants. Likewise, neither age nor education level was a significant predictor of the Q6 responses. In particular, the FU visit type demonstrated a strong link with a higher Q6 response rate, as evidenced by the coefficient of 0.241 (95% CI 0.059 to 0.423; *P*=.01). However, no significant association was identified between the diagnostic categories and Q6 response (both *P*>.05). When evaluating the multicollinearity model, the variance inflation factors ranged from 1.01 to 1.29, with an average of 1.15, confirming that it was not a factor of concern.

**Table 2:** Characteristics of FV^a^ and FU^b^ patients.

Characteristics		FV (n=100)		FU (n=100)	Total (N=200)		*P* value^c^	
Age (y), mean (SD)		42.52 (13.80)		54.35 (23.04)	48.44 (19.85)		<.001	
**Questionnaire results^d^, mean (SD)**								
	Q1	4.01 (0.66)	4.01 (0.61)		4.01 (0.63)	>.99		
	Q2	4.08 (0.66)	4.11 (0.72)		4.09 (0.69)	.71		
	Q3	4.16 (0.79)	4.23 (0.62)		4.20 (0.71)	.49		
	Q4	4.00 (0.67)	4.02 (0.67)		4.01 (0.66)	.83		
	Q5	4.42 (0.57)	4.39 (0.51)		4.41 (0.54)	.70		
	Q6	4.05 (0.66)	4.29 (0.57)		4.17 (0.63)	.006		
	Q7 BC^e^	3.21 (0.59)	3.10 (0.48)		3.15 (0.54)	.15		
	Q7 AC^f^	4.08 (0.58)	3.91 (0.59)		4.00 (0.59)	.04		
**Sex, n (%)**								.89
	Female	61 (61)	60 (60)		121 (60.5)			
	Male	39 (39)	40 (40)		79 (39.5)			
**Education, n (%)**								.25
	Below university	36 (36)	44 (44)		80 (40)			
	University and above	64 (64)	56 (56)		120 (60)			
**Diagnostic category^g^, n (%)**								.38
	Arthropathies	67 (68.4)	77 (77)		144 (72.7)			
	CTD^h^ or vasculitides	20 (20.4)	16 (16)		36 (18.2)			
	Other	11 (11.2)	7 (7)		18 (9.1)			

^a^FV: first visit.

^b^FU: follow-up.

^c^*P* values from *t* tests (continuous variables) and chi-squared tests (categorical variables).

^d^Q1-Q7 refer to the questionnaire items that assess patient attitudes.

^e^BC: before the consultation.

^f^AC: after the consultation.

^g^The diagnosis categories assigned by rheumatologists.

^h^CTD: connective tissue diseases.

**Table 3:** Variables and values used in the regression.

Variable	Value
Sex	0=female, 1=male
Age (y)	Primary value
Visit	0=FV^a^, 1=FU^b^ visit
Education	0=below university, 1=university and above
Diagnostic category	0=arthropathies, 1=connective tissue diseases and vasculitides, 2=others

^a^FV: first visit.

^b^FU: follow-up.

**Table 4:** Multivariate linear regression analysis for Q6^a^.

Variable			Coefficient (95% CI)		*P* value
Sex			0.066 (–0.115 to 0.247)		.47
Age (y)			0.000 (–0.004 to 0.005)		.86
Visit			0.241 (0.059 to 0.423)		.01
Education			0.084 (–0.098 to 0.266)		.36
**Diagnostic category**					
	Connective tissue diseases and vasculitides	0.097 (–0.138 to 0.333)		.42	
	Others	0.162 (–0.148 to 0.472)		.31	

^a^Q6: question 6 in Table 1.

## Discussion

### Principal Findings

In a cross-sectional study framed by the RE-AIM framework, we assessed the user satisfaction and acceptability of a health care chatbot among 200 patients, exploring potential variations across demographic strata, such as age, sex, and education. Our cohort, comprising 60.5% female participants, mirrored the demographics in Singapore [36]. Overall, our analysis revealed a favorable reception for the chatbot, as reflected by mean scores nearing 4 on a 5-point scale for most questionnaire items related to information retrieval and diagnosis via the chatbot.

### Effect of Interaction With the Physician

We also aimed to elucidate how FV and FU patients, representing various stages of treatment, engaged in and viewed the chatbot. The results revealed key differences between the groups. Specifically, FU patients reported a significantly higher willingness to reuse the chatbot for symptom investigation than FV patients. This suggests that FU patients may become more receptive to chatbots for symptom analysis after gaining familiarity with their clinical context through ongoing care. Further, the survey indicated a favorable shift in patients’ attitudes toward the acceptability of chatbot-based diagnosis after interacting with physicians. Patients expressed greater openness to the chatbot and made a diagnosis based on their symptoms after the clinical encounter. This highlights that physician endorsements may help mitigate the initial concerns patients have regarding the chatbots’ diagnostic accuracy. These findings align with prior studies demonstrating that health chatbot acceptability can be affected by factors such as perceived accuracy of diagnosis, inability to conduct physical examinations, and patient preference for communication with physicians over chatbots [37,38]. Our observations imply that establishing patient confidence in the legitimacy of chatbots as diagnostic decision-making aids relies heavily on securing ongoing support and reassurance from health care providers as part of the care team [11].

### Effect of Age and Sex

We found that patients in all age groups had a similar acceptance of using a chatbot to search for information and as a diagnostic tool. Similarly, sex did not significantly influence the chatbot’s acceptability. These results suggest that chatbots could benefit patients at different stages of treatment as both a source of information and a diagnostic tool. Our findings align with those of a recent study by Iancu and Iancu [23], who found that age and sex did not appear to influence chatbot use, whereas subjective norms, health consciousness, and perceived convenience were influential factors. Furthermore, Chang et al [29] found that attitudes and subjective norms were positively related to individuals’ intentions to use medical chatbots. Taken together, it is evident that to maximize the adoption and use of chatbots, designers and developers should adopt user-centered approaches that address user concerns and problems [39].

### Limitations

Despite these insightful findings, this study had several limitations. The participant pool, mainly drawn from clinical settings, such as new referrals and FUs at rheumatology clinics, may not fully represent a broader population. The chatbot’s design, catering to Asian patients diagnosed with AIIRDs, may limit the generalizability of the results. Although structured, the RE-AIM model brought about challenges owing to its inherent intricacies. Moreover, the sampling process might have introduced a nonresponse bias, with only 200 (16.2%) of the 1232 patients providing consent to participate, which necessitates consideration. This study also overlooked the specific challenges faced by 31 participants aged >71 years. Although this group reported no explicit difficulties, the broader context of Singapore’s digital literacy programs aimed at seniors may have been a contributing factor.

### Implications

For a more comprehensive understanding of patient acceptance of chatbots, future research could also delve into the socioeconomic and cultural factors that influence chatbot adoption, possibly by referencing broader digital literacy initiatives. Exploring strategies to enhance patient engagement, possibly through gamification or personalized user experiences, could also be considered in subsequent studies. Moreover, addressing challenges such as data privacy and the complexity of medical contexts is crucial for the integration of chatbots in health care. Multicenter studies with larger sample sizes are necessary to refine the accuracy and representativeness of our findings. Transitioning to machine learning–driven prediction models may address concerns regarding chatbot diagnostic accuracy [40]. Moreover, an education and training framework for health care providers encompassing informational sessions and hands-on training modules is essential for successful chatbot integration into health care practice. Finally, establishing standardized guidelines and fostering an iterative improvement process based on provider feedback can significantly contribute to the refinement and broader acceptance of chatbot interfaces in health care settings [41,42].

### Conclusions

This study underscores the potential of chatbots, particularly those tailored for AIIRDs, to affect rheumatology care delivery positively. Our results revealed that patients who had FU visits showed a stronger propensity to re-engage with the chatbot, particularly when they were offered information on symptomatology. Sex, educational attainment, and specific diagnostic categories did not significantly influence chatbot acceptance. Furthermore, greater comfort with chatbot-mediated diagnoses was observed after physician consultations. This suggests that prior clinical experience and physician validation can significantly improve confidence in emerging digital tools. Although more rigorous investigations are warranted to fine-tune their integration into routine care, current evidence suggests that appropriately developed chatbots can fortify the nexus between patients and health care professionals, thereby improving the overall efficacy of rheumatology care.

## Appendix

Multimedia Appendix 1 Comprehensive collection of questionnaire responses segregated by sex, education level, and diagnostic categories, alongside visuals of the chatbot interface.

## Abbreviations

AI: artificial intelligence
AC: after the consultation
AIIRD: autoimmune inflammatory rheumatic disease
BC: before the consultation
FU: follow-up
FV: first visit
RE-AIM: Reach, Effectiveness, Adoption, Implementation and Maintenance

## Data Availability

The data underlying this paper are available in the paper and web-based Multimedia Appendix 1.
