# Supplementary material for: Patient Acceptability of Symptom Screening and Patient Education Using a Chatbot for Autoimmune Inflammatory Diseases: Survey Study
Source: JMIR Form Res. 2023 Dec 28;7:e49239. doi: 10.2196/49239 (PMC11019963; doi:10.2196/49239)

**Table S1.** Questionnaire responses by sex.

| Question | Female | Male | p-value |
| --- | --- | --- | --- |
| Q1 | 4.04 (0.64) | 3.96 (0.63) | 0.389 |
| Q2 | 4.12 (0.69) | 4.05 (0.70) | 0.465 |
| Q3 | 4.17 (0.70) | 4.23 (0.72) | 0.597 |
| Q4 | 3.96 (0.66) | 4.09 (0.66) | 0.177 |
| Q5 | 4.40 (0.54) | 4.42 (0.55) | 0.789 |
| Q6 | 4.15 (0.64) | 4.20 (0.61) | 0.555 |
| Q7 BC | 3.17 (0.51) | 3.13 (0.59) | 0.550 |
| Q7 AC | 3.93 (0.56) | 4.09 (0.62) | 0.069 |

The p-values were obtained by independent samples t-tests comparing the mean scores.

**Table S2.** Questionnaire responses by education level.

| Question | Below University | University degree or above | p-value |
| --- | --- | --- | --- |
| Q1 | 3.96 (0.60) | 4.04 (0.65) | 0.388 |
| Q2 | 4.11 (0.64) | 4.08 (0.73) | 0.771 |
| Q3 | 4.25 (0.75) | 4.16 (0.67) | 0.370 |
| Q4 | 4.01 (0.67) | 4.01 (0.67) | 0.966 |
| Q5 | 4.38 (0.54) | 4.42 (0.54) | 0.523 |
| Q6 | 4.13 (0.66) | 4.20 (0.60) | 0.409 |
| Q7 BC | 3.11 (0.57) | 3.18 (0.52) | 0.365 |
| Q7 AC | 3.99 (0.56) | 4.00 (0.61) | 0.884 |

The p-values were obtained by independent samples t-tests comparing the mean scores.

**Table S3.** Responses to questionnaires by diagnostic category.

| Question | Arthropathies | CTD/vasculitides | Other | p-value |
| --- | --- | --- | --- | --- |
| Q1 | 4.01 (0.59) | 4.03 (0.77) | 4.00 (0.77) | 0.982 |
| Q2 | 4.09 (0.67) | 4.08 (0.77) | 4.11 (0.76) | 0.990 |
| Q3 | 4.19 (0.71) | 4.17 (0.70) | 4.22 (0.73) | 0.961 |
| Q4 | 4.05 (0.64) | 3.89 (0.75) | 3.94 (0.73) | 0.401 |
| Q5 | 4.40 (0.52) | 4.36 (0.64) | 4.50 (0.51) | 0.675 |
| Q6 | 4.15 (0.62) | 4.22 (0.68) | 4.28 (0.57) | 0.648 |
| Q7 BC | 3.13 (0.56) | 3.17 (0.51) | 3.28 (0.46) | 0.551 |
| Q7 AC | 3.98 (0.58) | 4.00 (0.63) | 4.00 (0.49) | 0.975 |

The p-values were obtained using a one-way ANOVA test to compare the mean scores between the diagnosis categories for each question.

**Figure S1.** Individual screenshot of the chatbot interface.


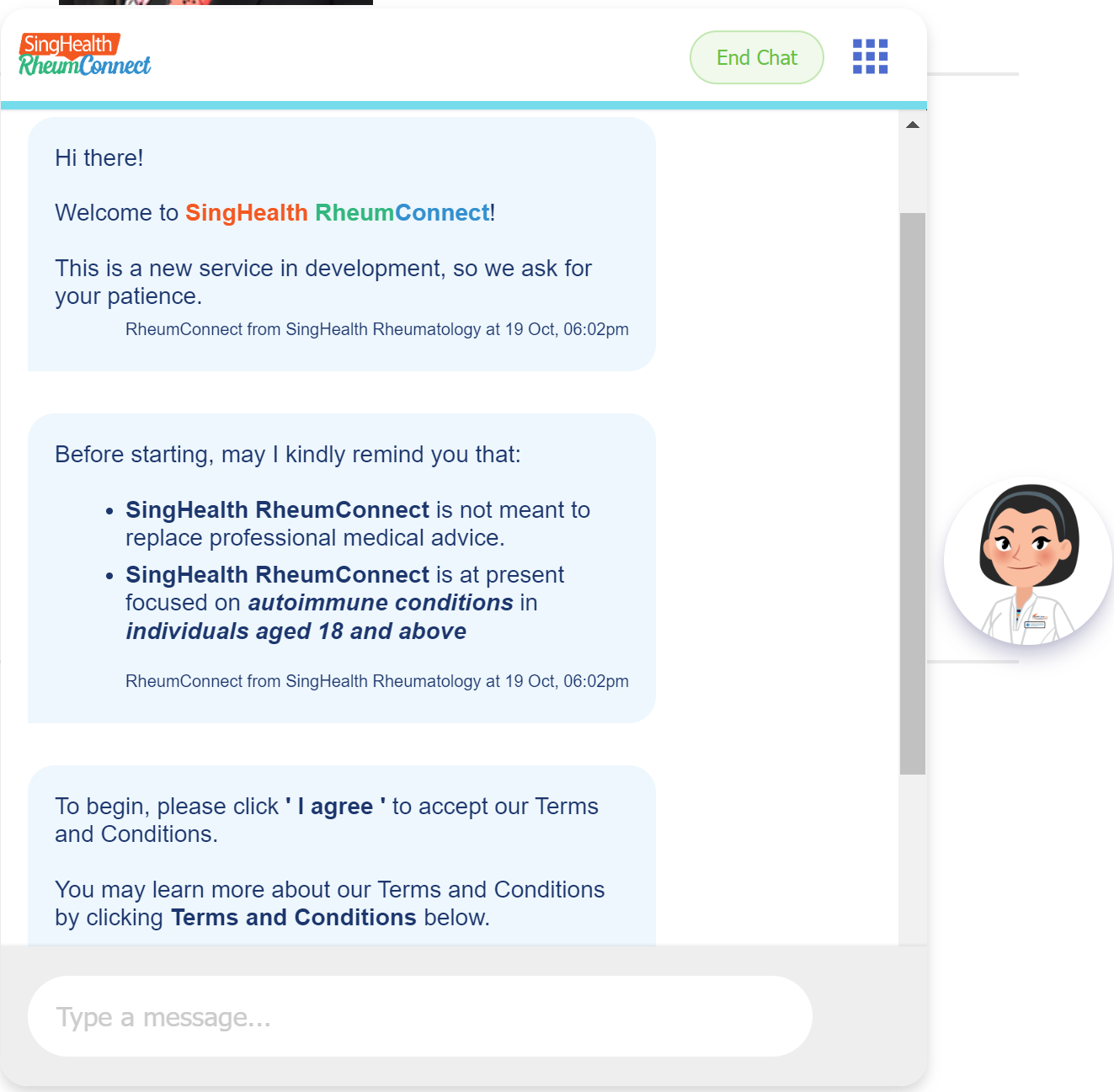


**Figure S2.** Individual screenshot of the chatbot interface.


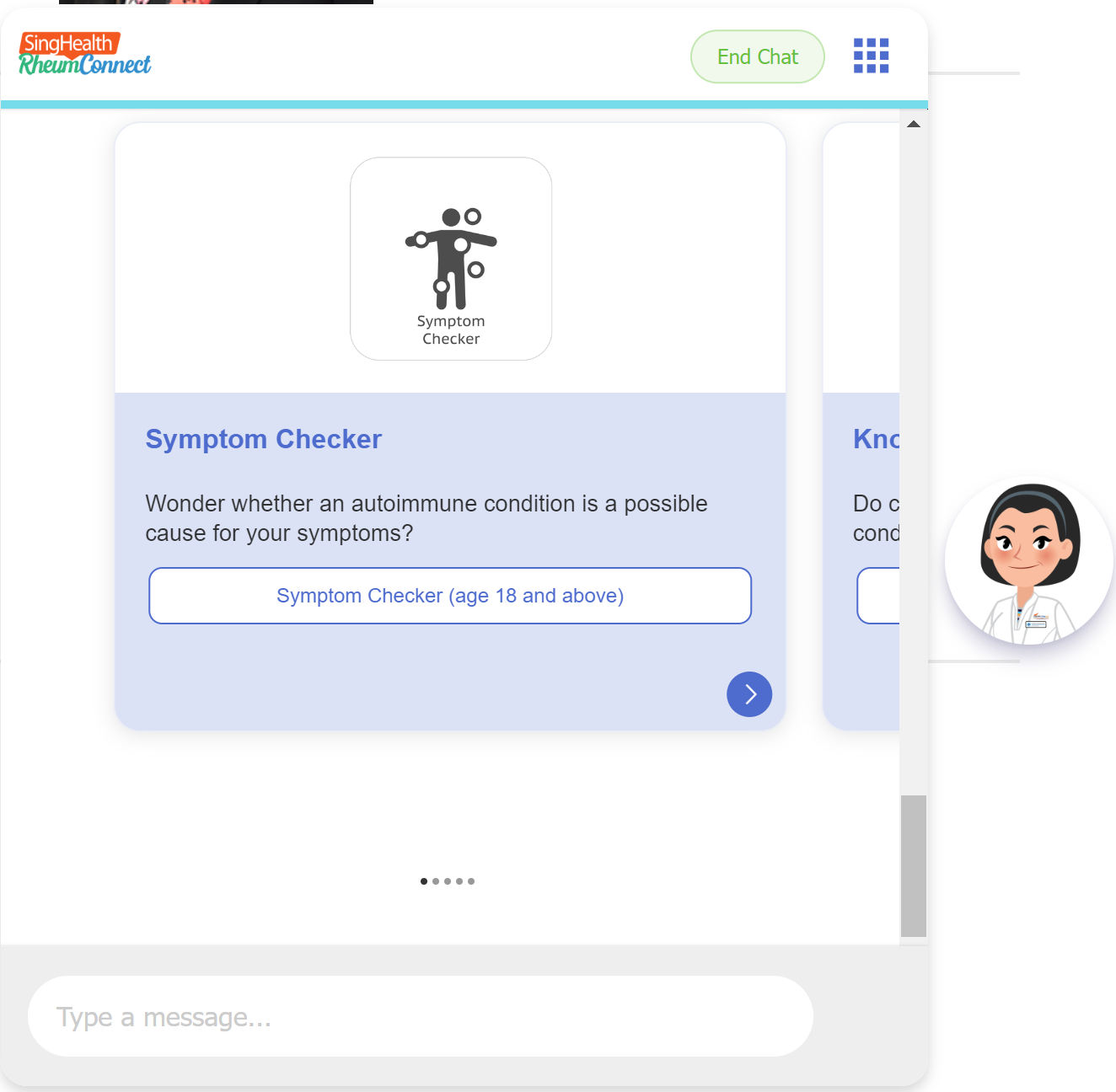


**Figure S3.** Individual screenshot of the chatbot interface.


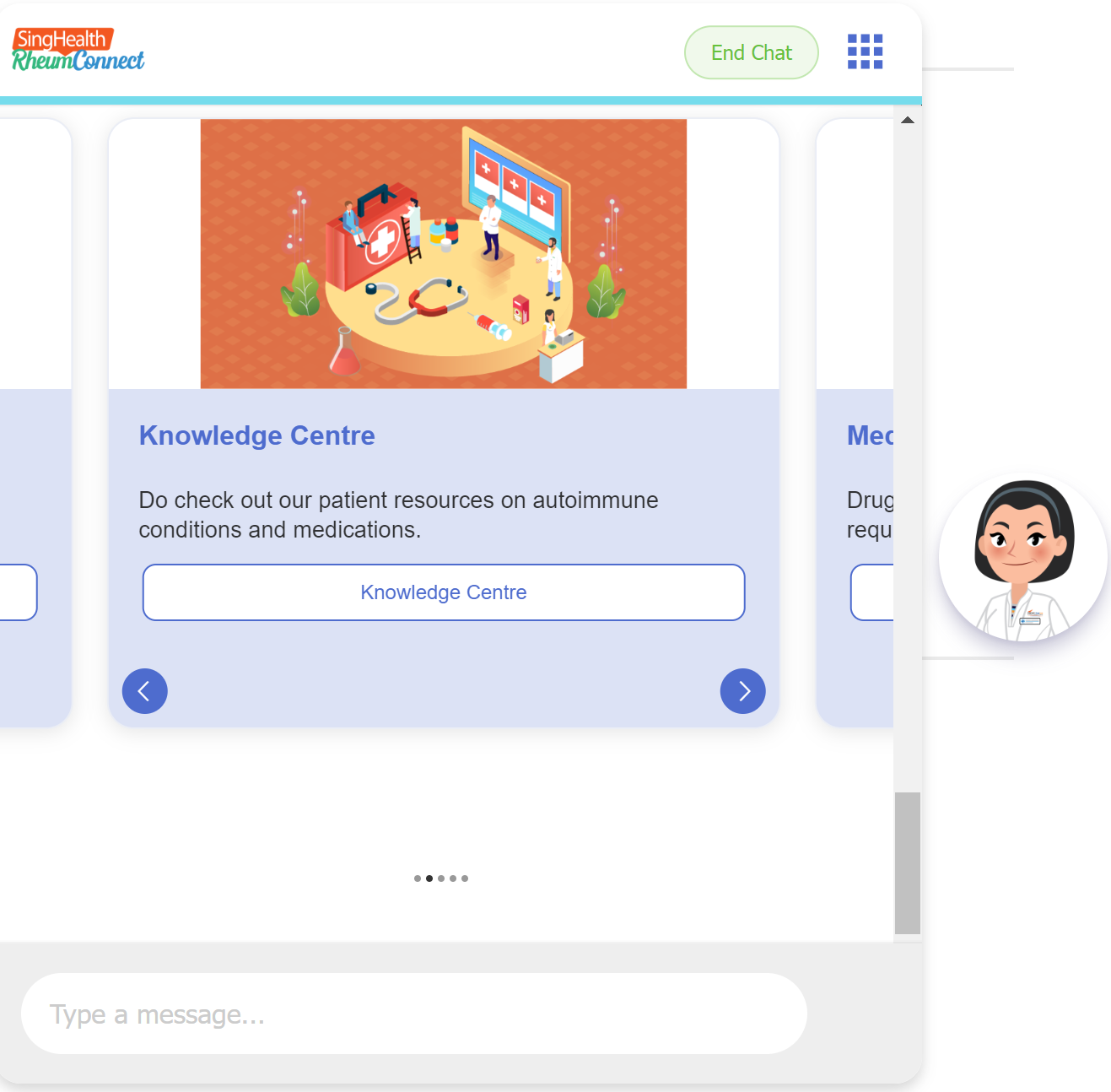


**Figure S4.** Individual screenshot of the chatbot interface.


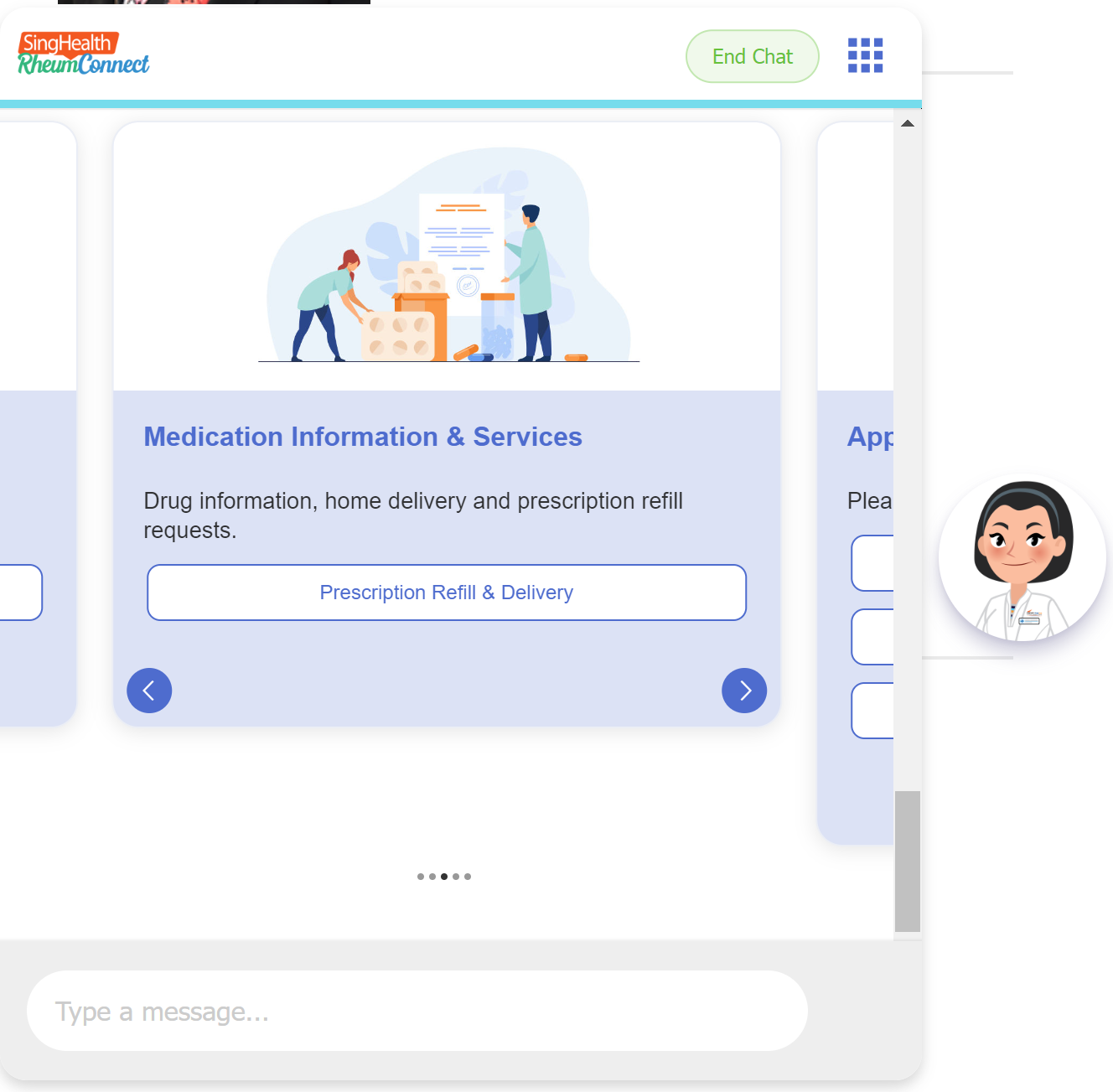


**Figure S5.** Individual screenshot of the chatbot interface.


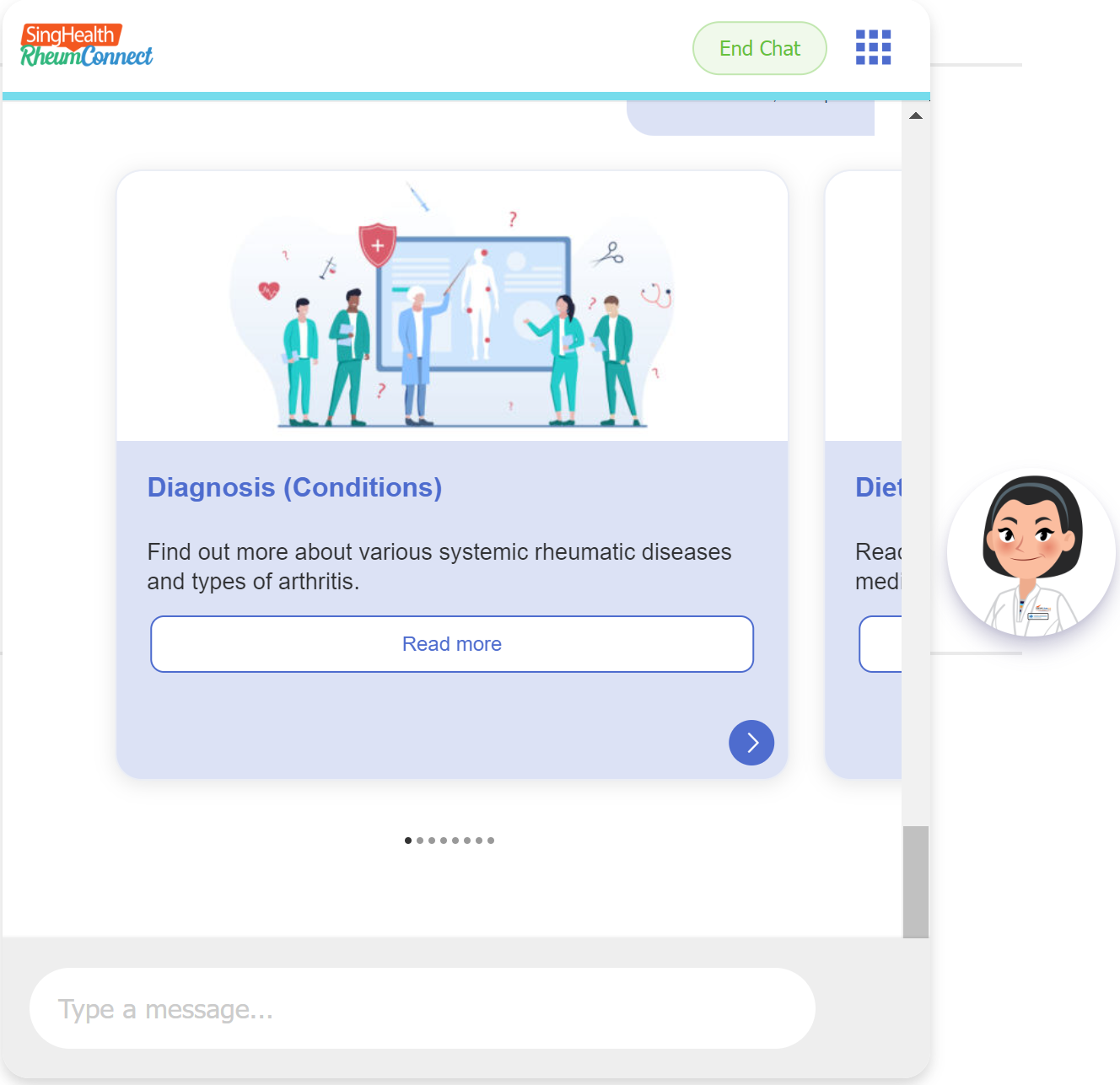


**Figure S6.** Individual screenshot of the chatbot interface.


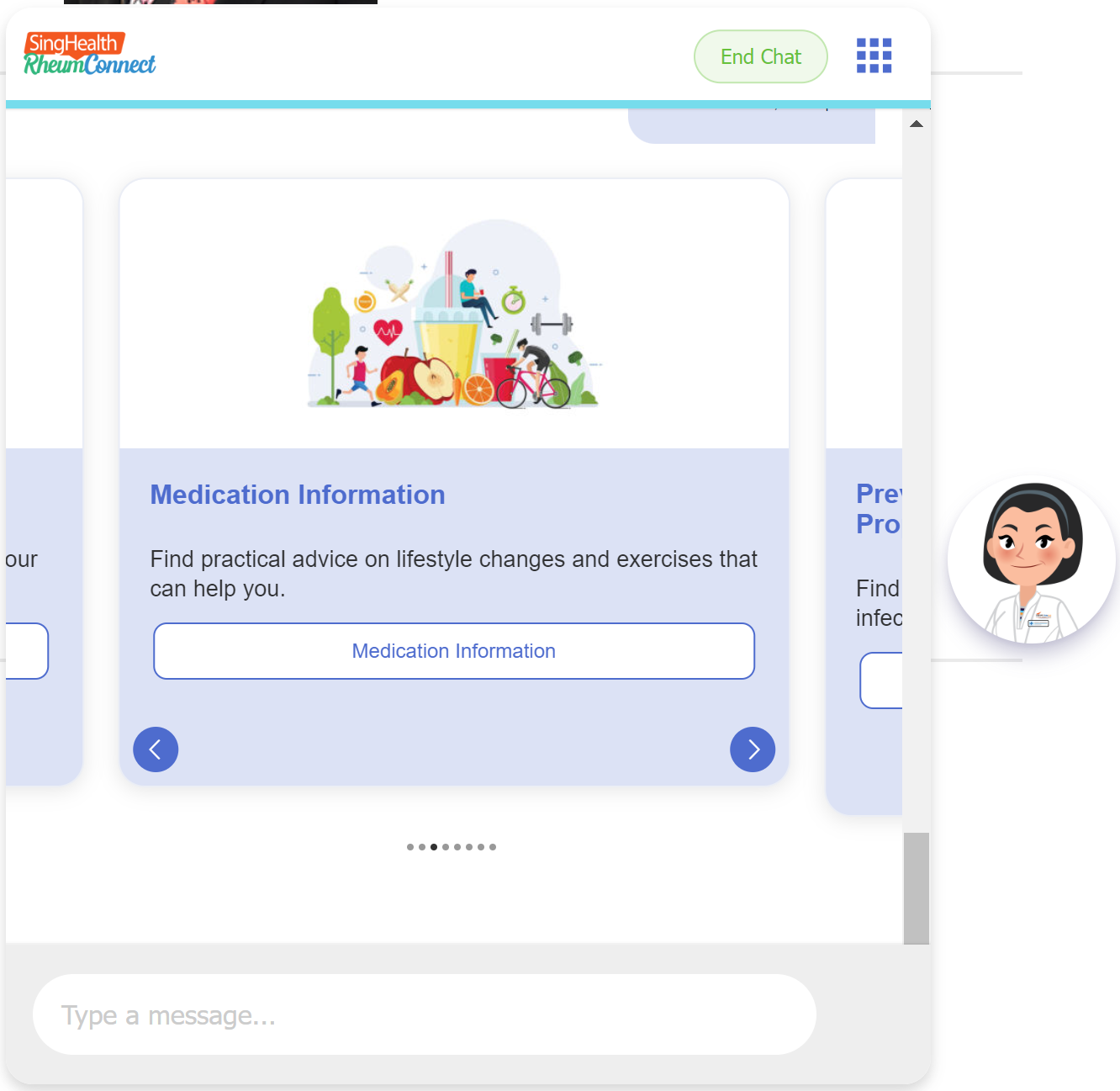


**Figure S7.** Individual screenshot of the chatbot interface.


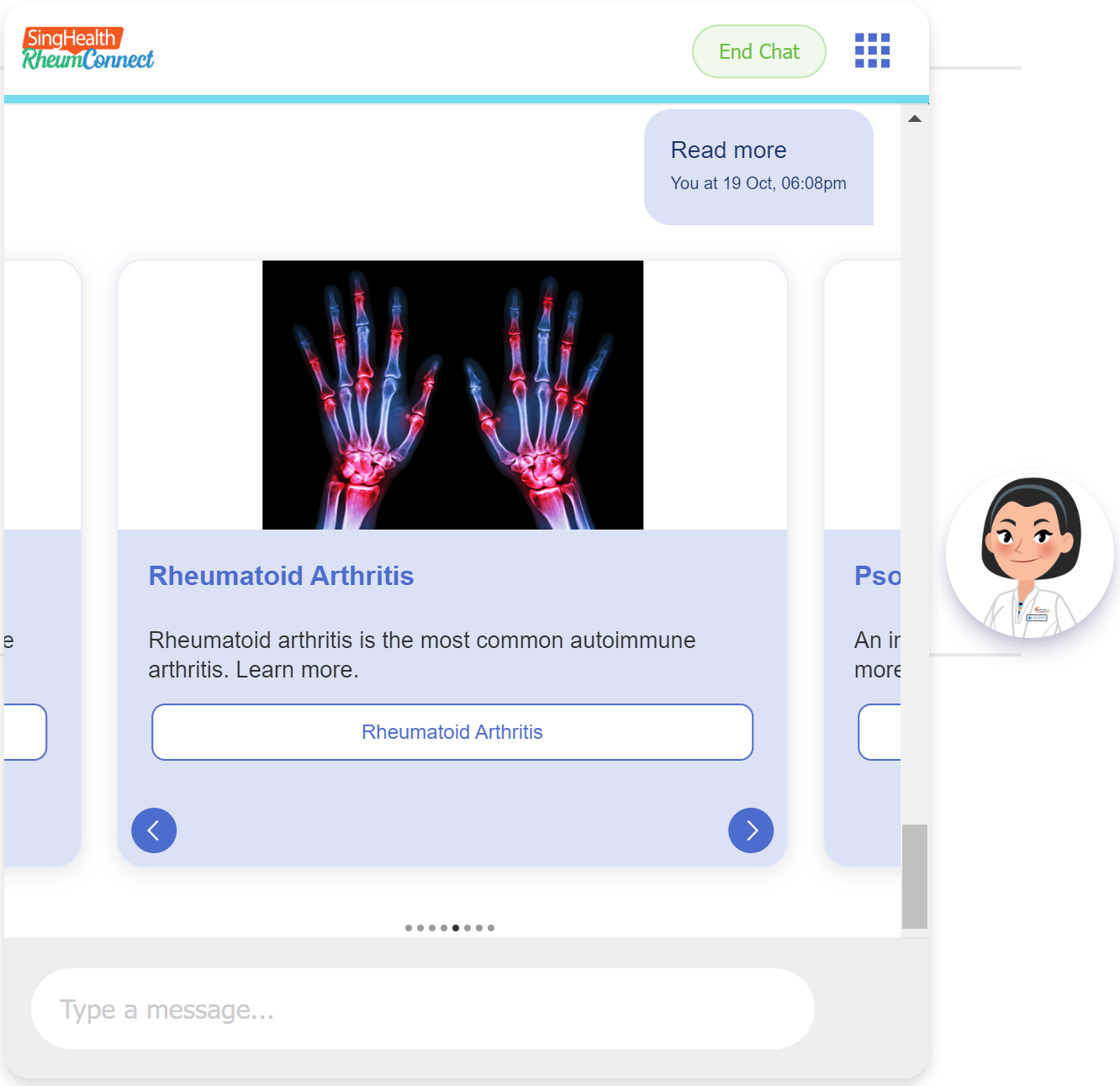

Supplement: Multimedia Appendix 1 [file formative_v7i1e49239_app1.docx]
